# Practical Notes on Diagnosis and Treatment

**Published:** 1908-05-30

**Authors:** 


### PRACTICAL NOTES ON DIAGNOSIS AND TREATMENT.

**Hyoscine in Obstetric Practice.**

Birchmore recommends hyoscine as the ideal anæsthetic for the accoucheur. It quiets the patient as satisfactorily as any narcotic and affords a practical anæsthesia of prolonged duration, and without harm either to mother or to child.

**Diphtheria and Antitoxin.**

Two axioms are well known—namely (1) That children require as large, or even larger, doses than adults, (2) that the earlier in the disease the injection is given the better the prognosis.—*Dr. H. Draper Bishop.*

**Medicinal Treatment of Rickets.**

To improve the tone of the alimentary canal give a couple of minims of tincture of nux vomica, 5 minims of tincture of rhubarb, and 3 or 4 grains of sulphocarbolate of sodium. Then cod-liver oil, especially in combination with hypophosphites, is the important medicine.—*Dr. G. A. Sutherland.*

**Ocular Paralysis in Influenza.**

Dr. K. A. Grossman records a case of influenza followed by paralysis of certain of the ocular muscles. The experience was repeated after a further influenzal attack. A peripheral neuritis is the presumed explanation. The same thing may occasionally be seen as the result of chronic alcoholism.

**Anti-Typhoid Inoculation.**

On the authority of Lieutenant-Colonel Leishman, R.A.M.C., it is stated that the 17th Lancers, the only regiment which has been exposed to a severe epidemic of enteric fever since the modified anti-typhoid vaccine has been in use, had 147 officers and men inoculated out of a strength of 509. In the epidemic there were 62 cases, with 11 deaths. All of the cases occurred among the uninoculated with the exception of two, and both of these were men who had refused the second inoculation. Further, both these cases recovered.

